# Excellent Outcomes in a Geriatric Patient with Multiple Brain Metastases Undergoing Surgical Resection with Cesium-131 Implantation and Stereotactic Radiosurgery

**DOI:** 10.7759/cureus.1970

**Published:** 2017-12-20

**Authors:** Sean S Mahase, Diana Julie, Susan C. Pannullo, Bhupesh Parashar, A. Gabriella Wernicke

**Affiliations:** 1 Radiation Oncology, NewYork-Presbyterian/Weill Cornell Medical Center; 2 Neurological Surgery, NewYork-Presbyterian/Weill Cornell Medical Center

**Keywords:** radiation oncology, brain metastases, stereotactic radiosurgery, cesium-131

## Abstract

Stereotactic radiosurgery (SRS) is a minimally invasive, focal treatment option for brain metastases. Multiple studies support its use in various settings as an effective, comparable alternative to surgery and whole-brain radiation therapy (WBRT). Here, we present excellent outcomes in a 90-year-old patient who underwent SRS after initially presenting at age 84 with multiple brain metastases of an unknown primary, as well as undergoing SRS to a site of tumor recurrence that was initially treated with surgical resection and intraoperative cesium-131 (Cs-131) brachytherapy. To our knowledge, this is one of the first reports describing the effective use of both intraoperative brachytherapy and SRS in the management of multiple brain metastases.

## Introduction

Brain metastases are the most prevalent central nervous system (CNS) tumors, developing in up to 40% of patients with cancer. Brain metastases portend a poor prognosis, and patients often succumb to a plethora of symptoms secondary to the tumor burden, including nausea and vomiting, mental status alterations, seizures, and focal neurological deficits [1]. Multidisciplinary efforts to address this increasingly prevalent burden have led to several therapeutic avenues whose application is based on a patient’s performance status, CNS, and extracranial disease burden, primary tumor site, and biology [1-2]. Surgical resection is a preferable option for solitary metastases, providing immediate relief from large, symptomatic lesions, as well as facilitating pathological diagnosis [1-3]. Whole-brain radiation therapy (WBRT) has been utilized in the post-operative setting, decreasing recurrence rates within the resection cavity, as well as the incidence of new metastatic foci within the brain parenchyma, but with no improvement in overall survival [4]. It is traditionally utilized as a primary modality when resection is not feasible in the setting of multiple metastases or in recurrent disease after initial hypofractionated radiation therapy (RT) to one or more sites. However, patients with longer survival are left to contend with well-documented neurocognitive impairments [1-4]. Other therapeutic options have shown promising results in selective settings, including the intraoperative application of high-dose permanent brachytherapy implants, such as iodine-125 (I-125) or cesium-131 (Cs-131), to improve local control [5].

The current therapeutic approach to brain metastases is in the midst of a paradigm shift with the refinement of stereotactic radiosurgery (SRS). Enabling high-dose radiation delivery with millimeter precision, SRS is applied as the primary therapeutic modality, as well as adjuvant therapy following surgical resection or WBRT. While initially reserved for patients with up to four brain metastases, numerous recent studies demonstrating comparable or improved outcomes compared with traditional treatments have culminated in National Comprehensive Cancer Network guidelines promoting SRS for multiple metastases, which notes that the total treatment volume is more prognostic for survival than the number of lesions [1]. The expanding role of SRS in brain metastases is attributed to technological advancements enabling a minimally invasive treatment option while preserving the quality of life (QoL). This is an especially valuable option for patients with poorer prognoses, such as poor performance status, radio-resistant histology, and older age [6-7]. Here, we present a case exemplifying QoL preservation in a geriatric patient undergoing SRS as a primary treatment modality for multiple new brain metastases, as well as at a site of tumor recurrence initially treated with surgical resection and intraoperative brachytherapy.

## Case presentation

**Figure 1:**
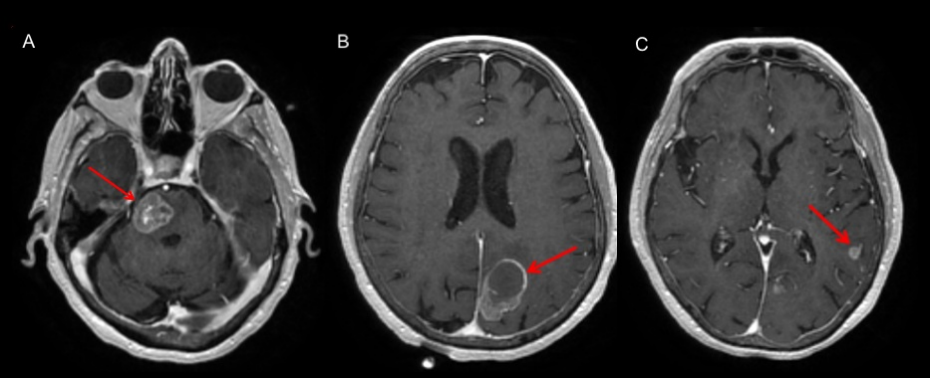
Representative MRI images at diagnosis, showing three brain lesions Axial images showing three heterogeneously enhancing lesions involving the (A) right pons, (B) the left inferior parietal lobe demonstrating a hemorrhagic component, and (C) the left supramarginal gyrus. MRI: magnetic resonance imaging

An 84-year-old female, never a smoker, with a history of hyperlipidemia and well-controlled hypertension, presented with sudden-onset dysgraphia, right lower and upper extremity weakness, and mild confusion. A brain magnetic resonance (MRI) scan with contrast revealed a 3.6 x 2.3 x 3.2 cm left inferior parietal lobe lesion with a hemorrhagic component; a heterogeneously enhancing 2.3 x 2.0 x 2.3 cm right pontine lesion extending to the anterior brachium pontis, and a 9 mm left supramarginal gyrus lesion (Figure 1). There was no evidence of midline shift or herniation. Her positron emission tomography (PET) scan showed a fluorodeoxyglucose (FDG)-avid left parieto-occipital mass and a right pontine mass, with no evidence of disease within the neck, chest, abdomen, or pelvis. These PET findings were corroborated with computerized tomography (CT) scans of the chest, abdomen, and pelvis, which were unremarkable for suspicious lesions or masses.

**Figure 2:**
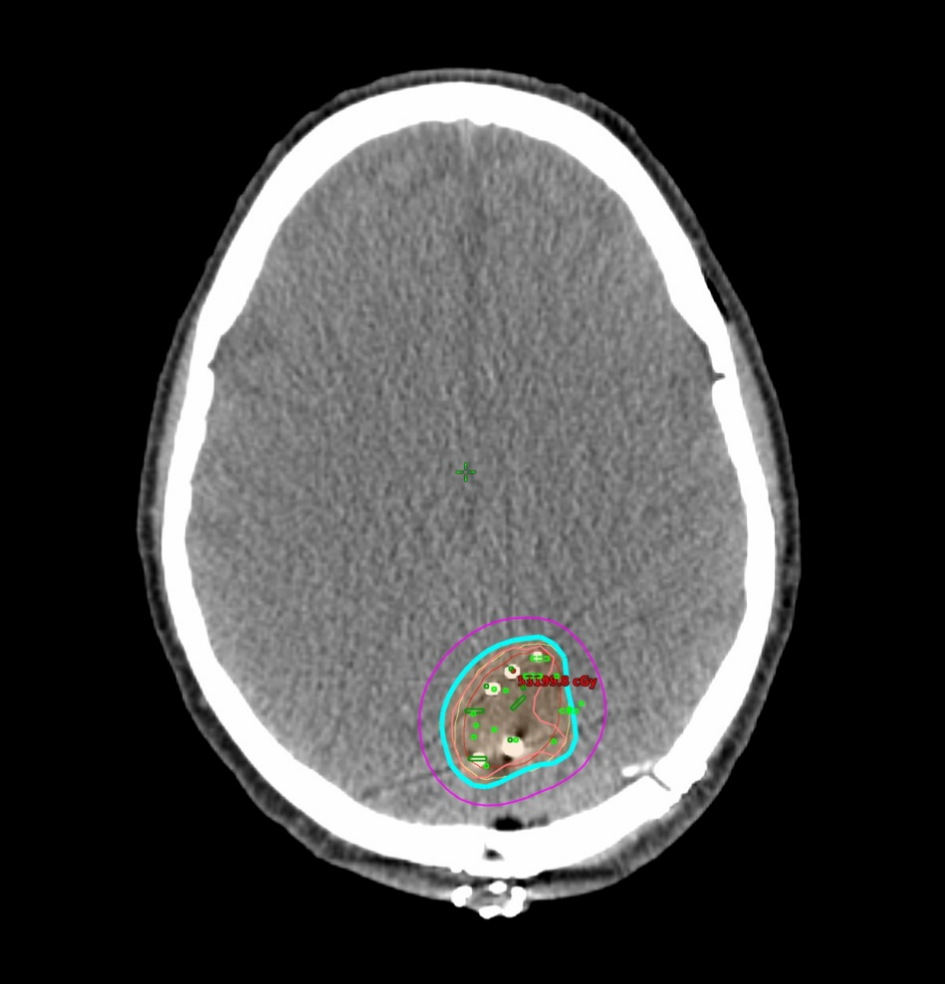
Cesium-131 implant dosimetry Cs-131 treatment plan. Isodose lines are as follows: Pink: 150% (120 Gy), Blue: 100% (80 Gy), Purple: 50% (40 Gy) Cs-131: Cesium-131

Following a discussion at the institutional multidisciplinary neuro-oncology tumor board, the patient underwent a left parietal craniotomy and resection of the left inferior parietal lobe lesion with an implantation of Cs-131 in the surgical cavity (after a frozen section pathological diagnosis confirming malignancy). The resected tumor was approximately 3.6 x 2.3 x 3.2 cm. A total of 22 Cs-131 seeds was used, with a delivered dose of 80 gray (Gy) to the 100% isodose line (Figure 2). The number of seeds used is determined by our institutional nomogram for volumetric Cs-131 implants. Immunohistochemistry revealed positive staining for cytokeratin seven (CK7), thyroid transcription factor one (TTF-1), and estrogen receptor (ER), and negative staining for cytokeratin 20 (CK20), progesterone receptor (PR), human epidermal growth factor receptor two (HER2-neu), caudal type homeobox two (CDX-2), Wilm's tumor one (WT-1), and epidermal growth factor receptor (EGFR), which was most consistent with metastases secondary to a non-small cell adenocarcinoma of the lung.

Due to the large size and location of the pontine lesion, a fractionated SRS using CyberKnife (Accuray Incorporated, California, United States) at an affiliated institution was recommended, with the patient undergoing 20 Gy in five fractions to the 83% isodose line. She also received 18 Gy in one fraction to the left supramarginal gyrus lesion using CyberKnife. The patient tolerated surgery and SRS well, without any significant acute or long-term side effects. She underwent both inpatient and outpatient physical therapy after her initial surgery and continued to live independently and lead an active life.

**Figure 3:**
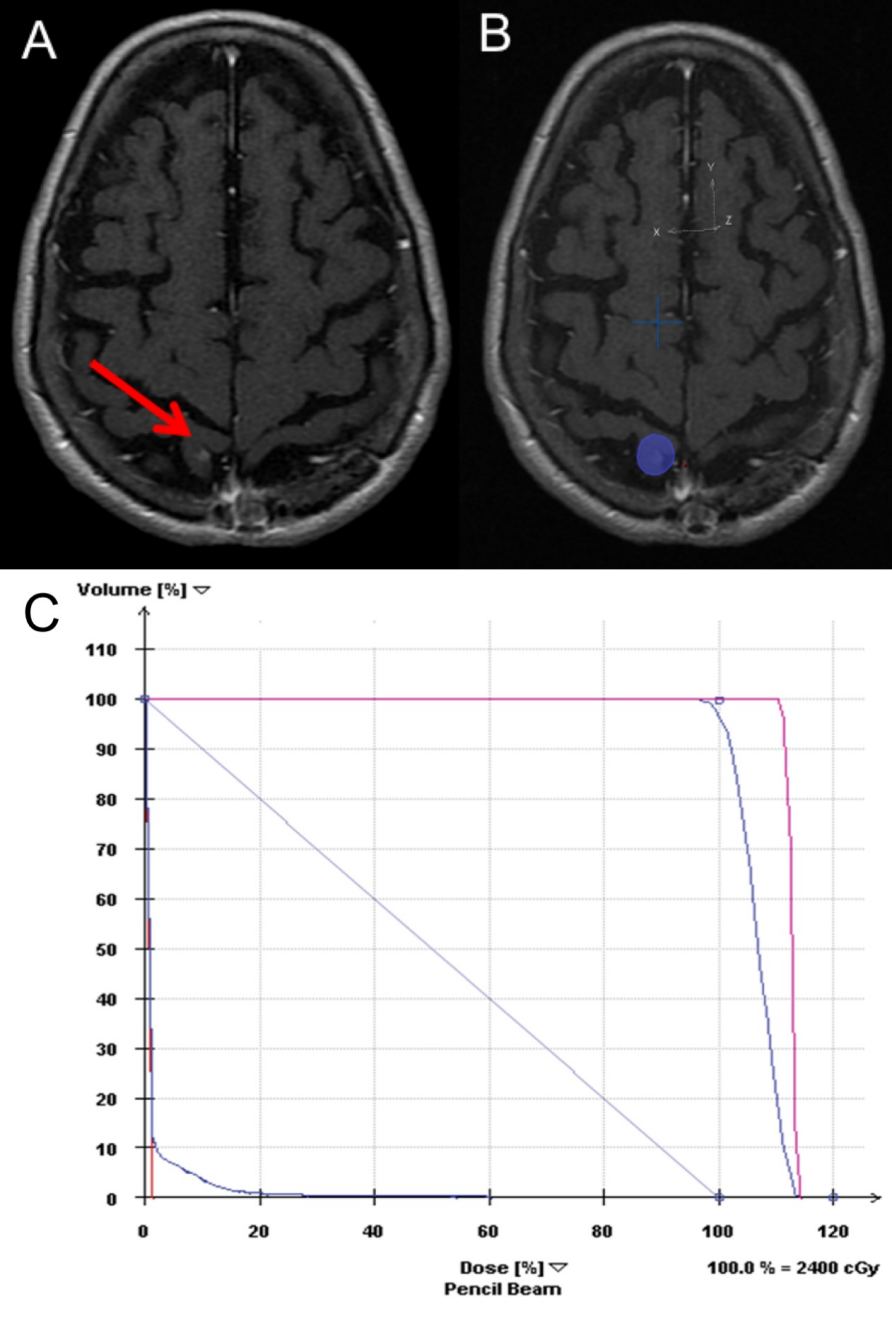
New postcentral gyrus lesion 1 year following initial treatment (A) Before treatment: 4 mm enhancing lesion in the right postcentral gyrus, (B) SRS plan, (C) DVH: Showing some of the doses to target regions (planning target volume in magenta and gross tumor volume in purple) and critical structures (right eye – green, left eye –red, left optic nerve – red, right optic nerve – green, right lens – teal, brainstem – green, optic chiasm – purple, normal brain tissue – light purple). DVH: dose volume histogram; SRS: stereotactic radiosurgery

One year later, a screening MRI of the brain with contrast revealed a new 4 mm enhancing lesion in the right postcentral gyrus. She underwent SRS to the right postcentral gyrus lesion: 24 Gy to the 84% isodose line in three fractions using a Varian iX linear accelerator (Varian Medical Systems, California, United States) (Figure 3).

**Figure 4:**
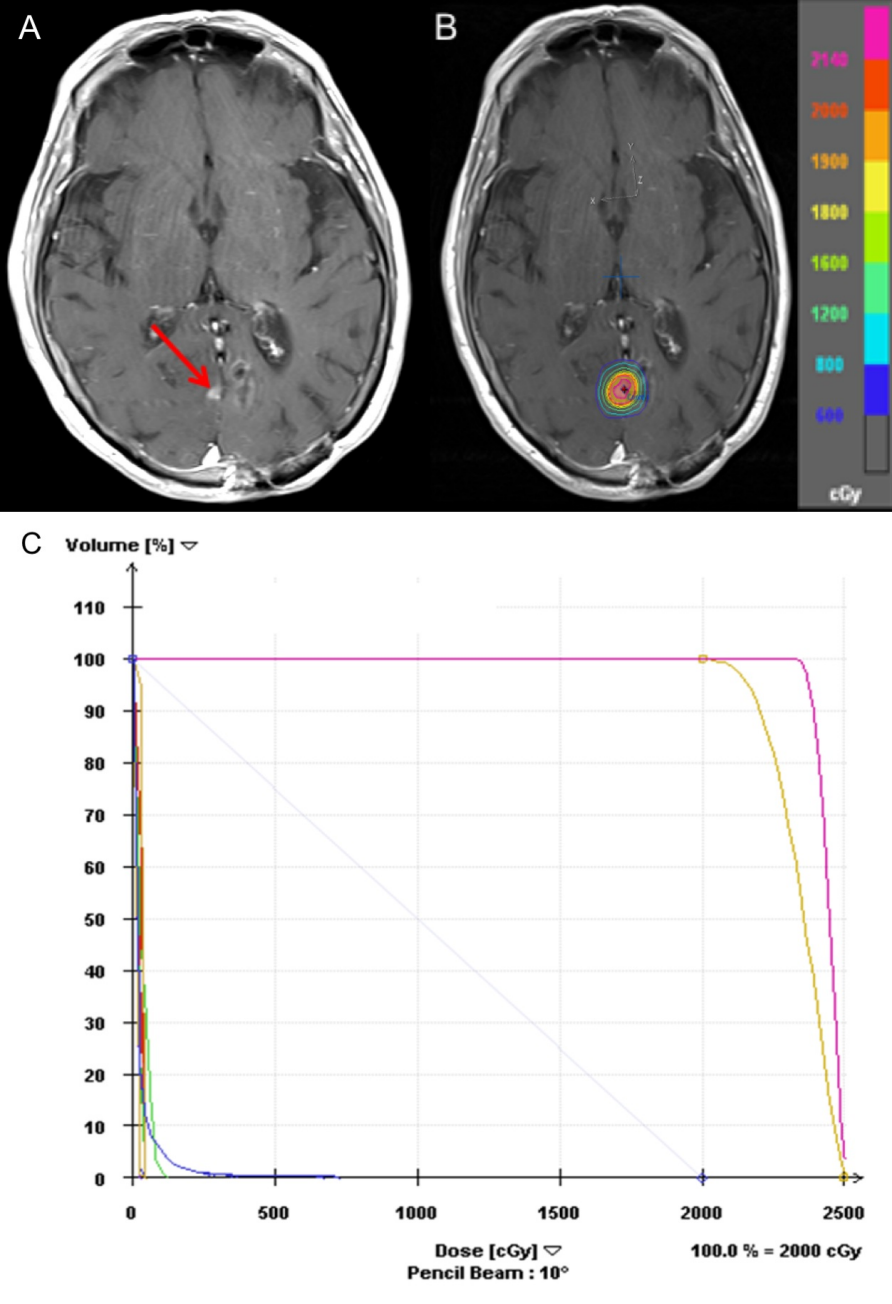
Recurrence in right occipital parasagittal lesion four years after resection (A) Before treatment: 6 x 5 mm nodule of enhancement to the right of the midline adjacent to the inferomedial aspect of the resection cavity suspicious for tumor recurrence, (B) SRS plan, (C) DVH: Showing some of the doses to target regions (planning target volume in magenta and clinical tumor volume in yellow) and critical structures (right eye – green, left eye – red, left optic nerve – red, right optic nerve –green, right lens – teal, brainstem – green, optic chiasm – purple, normal brain tissue – blue) DVH: dose volume histogram; SRS: stereotactic radiosurgery

Interval studies were unremarkable until four years following her initial presentation (88-years-old at this time point), when a routine surveillance MRI revealed a 6 x 5 mm nodule of enhancement to the right of the midline, approximately 6.5 mm adjacent to the inferomedial aspect of the resection cavity. There was a mild degree of vasogenic edema adjacent to this new lesion in the parasagittal right occipital lobe. As this finding could be attributed to tumor recurrence, or to delayed treatment effect, it was decided to perform a follow-up MRI three months after this initial scan, which showed an increase in the aforementioned nodular focus of enhancement. This was concerning for regional recurrence, which was defined in our institution's Cs-131 guidelines as the presence of a new nodular or contrast enhancement greater than 5 mm from the resection cavity on MRI [5]. She underwent SRS, 20 Gy in one fraction, to the 80% isodose line to the right occipital parasagittal lesion using a Varian iX linear accelerator (Figure 4).

**Figure 5:**
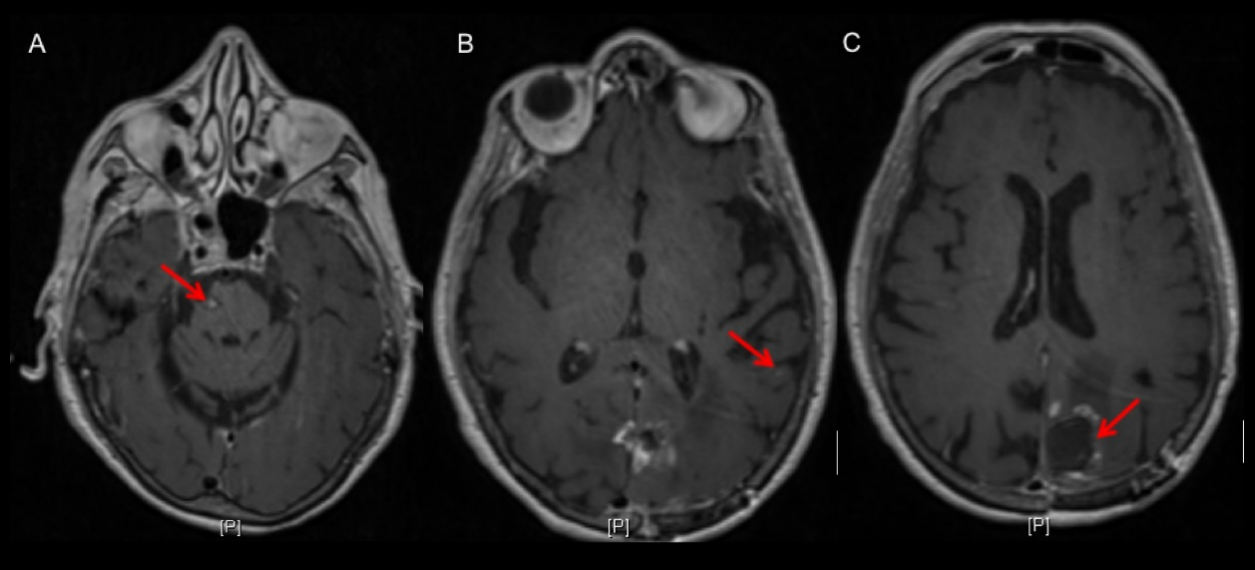
Representative MRI images at patient’s last follow-up Axial images showing stable, small enhancing lesions corresponding to previously treated metastases in the (A) right lateral pons and (B) left supramarginal gyrus. (C) Stable appearance of the left parieto-occipital resection site status post craniotomy and brachytherapy for the resection of a metastasis six years ago and subsequent SRS two years ago for tumor recurrence. SRS: stereotactic radiosurgery

The patient’s most recent MRI (six years after initial diagnosis) showed stable irregular/nodular enhancing lesions in the right lateral pons, the left parietal lobe at the supramarginal gyrus, and the left parieto-occipital white matter; all consistent with edema and/or treatment effects (Figure 5). There were no new lesions identified on imaging. The patient is now 90-year-old and still living an active life. At her last follow-up, she was noted to walk with a walker but had good balance. She had symmetrical four out of four strength bilaterally in her upper and lower extremities and was without focal neurological deficits.

## Discussion

This case highlights several clinical concepts commonly considered in the management of brain metastases with SRS. The patient's overall excellent performance status, symptomatic presentation, and need for establishing a histological diagnosis were all factors favoring the resection of her dominant lesion [1-2]. The multidisciplinary decision to consolidate her surgical resection with focal therapy rather than WBRT is representative of a current paradigm shift in treatment approaches. The smaller right pontine lesion and left supramarginal gyrus lesions, as well as the right post-central gyrus lesion that appeared one year later, were all definitively treated without adverse side effects and without recurrence.

Recurrence rates following surgical resection alone approach 46%, advocating adjuvant measures to improve local control. Intracavitary brachytherapy with Cs-131 was selected to offer an immediate RT to the resection bed, avoiding tumor cell repopulation in the interval between surgery and adjuvant treatment, while conformal seed placement confers adequate dosing to the irregularly-shaped resection bed [5,8]. Cs-131 was chosen over I-125 due to a plethora of favorable characteristics: while I-125 has a half-life of 60 days, Cs-131 has a half-life of 9.69 days, thus 90% of the dose will be absorbed in approximately 33 days, enabling earlier initiation of adjuvant treatments and reducing potential radiation exposure in the event that a subsequent neurosurgical procedure was required. Additionally, previous studies report low rates of radiation necrosis with Cs-131, a major concern when employing I-125 [5]. SRS is also an appealing post-operative option. While round targets are ideal for SRS planning (as irregular-shaped cavities can complicate conformal planning), intensity-modulated radiation-therapy-based SRS may enable higher conformal treatment to irregularly shaped resection cavities. Intraoperative brachytherapy is another favorable option, enabling a high-dose RT to a conformal treatment area during the time of surgery. Unfortunately, our patient developed a regional recurrence at the site of her resection, which was successfully treated with SRS. A recent study on the clinical outcomes of brain metastases treated with resection and intraoperative Cs-131 brachytherapy reported regional recurrences in three of 46 treated tumors, all of which were successfully treated with SRS at doses of 18 to 25 Gy [5]. Several studies report a greater preoperative tumor diameter to be a significant predictor of local failure and radionecrosis in postoperative SRS [5]. A prospective randomized trial comparing postoperative SRS to interoperative brachytherapy is warranted to best compare local control and radionecrosis rates between these two treatment modalities. 

A single institution retrospective analysis reported 36% of 342 patients with newly diagnosed brain metastases had an undiagnosed primary site. Among this 36%, further evaluation revealed a primary lung lesion in 60% of the cases and non-lung primaries in 14% of the cases. Notably, 26% of primaries remained unknown [9]. The authors recommended a brain lesion biopsy for identification if a primary site was not otherwise found. While a histological analysis of our patient’s resected lesion was consistent with a lung primary, interval scans spanning six years after her initial diagnosis failed to detect a primary extracranial lesion. However, studies suggest that there is no survival difference among those with diagnosed and undiagnosed primary lesions. Conversely, performance status, tumor burden, treatment response, and age are stronger prognostic indicators of overall outcomes [3,7,9].

A more traditional approach to our patient may have been surgical resection followed by WBRT [1,4,7]. However, the inherent benefits of WBRT must be weighed against the long-term neurological effects in an expanding geriatric community consisting of a growing number of high-functioning individuals, and it might be especially detrimental among those with pre-existing cognitive impairment. A retrospective review of the ‘elderly’ (age 70-79 years) and the ‘very elderly' (age ≥ 80 years old) patients receiving WBRT (30 – 37.5 Gy in 10-15 fractions) or SRS (15 – 24 Gy in one fraction; 18-24 Gy in three fractions; 25 Gy in five fractions) for newly diagnosed brain metastases reported a median overall survival of 4.3 months among the WBRT cohort and 14.4 months in those treated with SRS. Additionally, on multivariate analyses including the intracranial disease burden and performance status, WBRT correlated with significantly higher rates of grades one through four toxicity. Notably, 51% of SRS patients underwent additional RT compared to 8% of the WBRT cohort, which could be attributed to the former having longer overall survival and, thus, more time to progress [6]. A recent, multicenter, randomized phase three trial comparing WBRT to SRS for resected brain metastasis found no difference in overall survival among these cohorts but noted a more-frequent decline in cognitive function with WBRT [10]. The current trend in brain metastasis management is moving toward local RT options.

Regarding our patient, during her initial presentation, both she and her daughter emphasized the importance of preserving her cognitive function to maintain overall her quality of life (QoL). Continuing to lead an active life at age 90, six years removed from her initial diagnosis and treatment and two years from her last SRS course, supports the use of SRS in the elderly to treat several lesions without the immediate inherent risks of neurosurgery or the long-term morbidity conferred by WBRT. Our management protocol using Cs-131 brachytherapy and the judicious use of SRS has resulted in maintaining the QoL of our patient while controlling her brain disease. This is a unique approach (using two types of highly conformal radiation technologies) and is not being followed in the majority of centers in the country or abroad. 

The efficacy of surgical resection in recurrent brain metastases has not been prospectively studied. There is also a dearth of level-one evidence regarding SRS in the salvage setting. In similar settings, WBRT has been reported in case series but is not widely used due to the potential side effects [4]. Repeat SRS to a previously treated site is a category 2B recommendation [1]. While level-two data have reported promising findings, it is difficult to delineate the impact of SRS versus intrinsic prognostic factors on outcomes [4]. Nevertheless, our case demonstrates that with careful selection, SRS is an effective and safe therapeutic modality in the setting of metastatic recurrence.

## Conclusions

In this report, we presented an 84-year-old patient with excellent performance status who was diagnosed with multiple symptomatic brain metastases of an unknown primary. She underwent surgical resection and Cs-131 brachytherapy to one lesion and SRS to the other two metastases. She later underwent additional courses of SRS to a fourth metastasis, as well as for a regional recurrence near the resection site. She tolerated her treatments with minimal short- and long-term side effects, is currently without new or recurrent sites of disease, and is leading an active life at age 90. To our knowledge, this is one of the first reports describing the effective use of both intraoperative brachytherapy and SRS in the initial management of multiple brain metastases, as well as the use of SRS for regional recurrence following intraoperative brachytherapy. Her case exemplifies diversely applying definitive SRS treatment in brain metastases with excellent long-term outcomes.
